# A Therapeutic Game for Sexually Abused Children and Adolescents (Vil Du?!): Exploratory Mixed Methods Evaluation

**DOI:** 10.2196/26062

**Published:** 2021-08-03

**Authors:** Joyce Johanna Endendijk, Henny Tichelaar, Menno Deen, Maja Deković

**Affiliations:** 1 Child and Adolescent Studies Utrecht University Utrecht Netherlands; 2 Studio Lapp Utrecht Netherlands

**Keywords:** child sexual abuse, psychotherapy, serious games, evaluation, working elements, acceptability

## Abstract

**Background:**

Talking about experiences of sexual abuse in therapy is difficult for children and adolescents. Possible reasons for this difficulty are a lack of vocabulary to describe the situation or feelings of shame, fear, and self-blame associated with sexual abuse. The serious game Vil Du?! was developed to help children open up about their sexual abuse experiences. Vil Du?! is a nonverbal communication game that resembles a dress-up game in which children can show the therapist what happened to them.

**Objective:**

This exploratory evaluation study examines which working elements of the game could be identified in therapy with victims of sexual abuse (aim 1). In addition, this study examines how therapists evaluate the acceptability of the game (aim 2).

**Methods:**

The therapists completed 23 web-based surveys on the use of Vil Du?! In addition, semistructured interviews were conducted with 10 therapists. The data were analyzed in NVivo following previously reported stepwise guidelines.

**Results:**

Regarding aim 1, therapists mentioned various working elements of Vil Du?!; for instance, Vil Du?! puts the child in control of the situation. In addition, Vil Du?! reduces barriers to disclosure because there is no need to talk or have eye contact with the therapist. Regarding aim 2, Vil Du?! was generally evaluated more positively than negatively by the therapists. For instance, therapists indicated that using Vil Du?! is time efficient and might make the treatment process less confronting and difficult for the client. According to therapists, most clients indeed experienced less tension and more positive (or neutral) emotions than negative emotions when using Vil Du?!

**Conclusions:**

The most important working elements of Vil Du?!, according to therapists, are that it enables children to regain control over their sexual abuse experiences and reduces barriers to disclosing sexual abuse experiences. The more positive evaluation of Vil Du?! indicates the acceptability of the game for therapists as well as their clients.

## Introduction

### Statement of the Problem

Talking about experiences of child sexual abuse (CSA) is difficult for children and adolescents. Possible reasons for this difficulty are feelings of shame, fear, and self-blame, a lack of understanding or denial of the situation, or a lack of vocabulary to describe the situation [1-4]. However, therapy for child and adolescent victims of CSA often relies heavily on verbal narration and the processing of one’s experiences. This is especially the case for cognitive behavioral therapy, which is the most often used and recommended form of psychotherapy for CSA [5,6]. To increase the suitability of cognitive behavioral therapy for children and adolescents, researchers have suggested the use of content that is tailored to the developmental needs of young clients and to emphasize elements of play [7,8]. Using play in a therapeutic setting puts the child or adolescent in control, which is known to reduce tension and stress [9]. In addition, play provides an age-appropriate and natural manner for children to express their feelings, which they are often unable to express through language [10]. Furthermore, play makes treatment more fun and engaging [11]. Originating from these ideas, the third author (M Deen) and others developed a serious game, called Vil Du?! [12], to help children open up about their sexual abuse experiences to their therapists. This study provides an exploratory evaluation of Vil Du?! among therapists using the game in therapy for sexually abused children or adolescents. More specifically, we examined the working elements of the game (ie, elements that can explain its effects on the therapy process) therapists identified, as well as therapists’ experiences with using the game (ie, benefits, limitations, and experiences of their clients).

### Other Serious Games for Children and Adolescents in Treatment for CSA

To date, there are limited serious game offerings for use in the context of CSA. One example is Orbit, a CSA prevention computer game targeted at students aged 8-10 years [13]. The goal of this adventure game is for the player to do everything they can to help the character Sammy that has suffered from sexual abuse. During several mini-games, children learn about recognizing CSA, perpetrator tactics, barriers to telling, building a healthy self-concept, and the importance of trusted adults who they can turn to. In addition, 2 unpublished master theses describe the development of serious prototype games aimed at helping children disclose CSA experiences [14,15]. Pharshy [14] developed a story-telling game in which children can create new stories of their own experiences, or edit existing stories, by using images, drawings, text, and self-created avatars. Parents or caregivers could monitor the child’s stories for possible CSA experiences. Andersson [15] developed a tool for use in a therapeutic context. The prototype contained different scenes and storylines that therapists could show and play out with children, which could spark conversations about the different (sexual) abuse-related situations children might find themselves in. The 3 existing games have been developed primarily for CSA prevention purposes and not for use in a CSA therapy context, such as Vil Du?! In addition, the effects of these 3 games have not yet been evaluated.

### Vil Du?! Design and Working Elements

**Figure 1:**
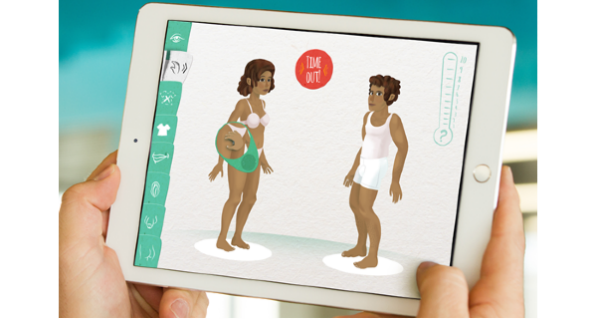
Overview of the game environment of Vil Du?!

**Figure 2:**
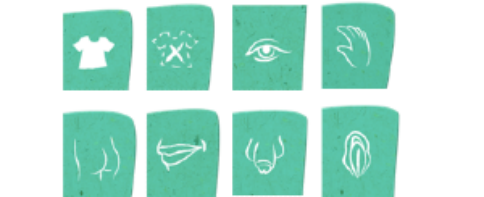
The icons that can be used in Vil Du?!

**Figure 3:**
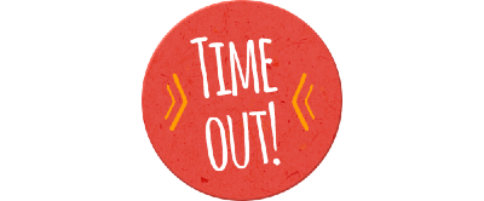
The time-out button in Vil Du?!

To the best of our knowledge, the serious game Vil Du?! (Danish for “Do you want to [talk about...]?!”) is the first digital game that is used in a treatment context for child and adolescent CSA victims. Vil Du?! is a nonverbal communication game in which children can show the therapist what happened to them (Figure 1). In the game, which resembles a *dress-up game*, both therapist and child operate a self-chosen character, each on their own tablet (provided by the therapist). The tablets are synchronized to each other, so actions performed on one screen are also visible on the other, enabling digital interaction between the therapist and child (without the necessity of looking each other in the eyes). Both players can perform various actions on the other character by clicking (eg, undress) or dragging explicit icons (eg, mouth, hand, penis, and buttocks; Figure 2) over the character’s body. Each player can express their boundaries or pause or stop the game by pressing a *Time-Out* button (Figure 3). While playing, the therapist can probe the child to talk about their experiences, thoughts, and feelings. In addition to the *Time-Out* button, Vil Du?! does not include verbal statements or textual markers. The goal of the game is to give children a voice without the need to talk and to put children in charge of creating their own story, normative structure, and values associated with love, sex, and romance. Vil Du?! has been used by therapists with children and adolescents between the ages of 5 and 17 [16].

The first phase in evaluating a new tool is to define the working elements of the intervention [17]. Hereby, the goal is to find and define promising practice-based elements or components that could explain what works in the use of a serious game in therapy for CSA victims [18]. These working elements refer to important elements through which serious games (ie, Vil Du?!) might lead to beneficial outcomes in therapy for CSA [19]. Several possible working elements are identified in the game. First, Vil Du?! might reduce barriers to disclosure of CSA experiences because of its nonverbal character and because the use of the game does not require face-to-face communication between the therapist and client. With Vil Du?!, children can show, instead of talk about, what happened to them, which might help to overcome the barrier of a lack of vocabulary to describe the situation [2]. In addition, there is no need for eye contact with the therapist when using Vil Du?! because the client can look at the tablet screen. This might reduce feelings of shame, fear, and self-blame associated with CSA experiences [1,3,4]. Researchers have indeed suggested that serious games “can be used as a ‘third party in the room’, helping to make the therapeutic process less difficult for adolescents by taking some of the emphasis off direct face-to-face conversations” [20].

Second, Vil Du?! provides children with an environment in which they can communicate through play. Vil Du?! resembles a dress-up game in which children can undress or redress a character and perform actions on, or with, the character by dragging icons over the character’s body (eg, use the hand icon to give a high five). The play element of Vil Du?! might have an effect on several components of therapy. For instance, Vil Du?! might be helpful for the cognitive restructuring of incorrect and maladaptive thoughts related to the CSA experience. CSA victims often have incorrect or maladaptive thoughts related to the abuse experiences (eg, *“*I could have prevented the abuse”). For therapists to restructure such incorrect and maladaptive thoughts, they first need to explicate children’s thoughts for which play (eg, with dolls, by drawing, or with Vil Du?!) can be used as a vehicle [21,22]. These explicit thoughts can be processed or restructured in successive therapy sessions. In addition, the explicit icons and characters of Vil Du?! enable children to engage in play that realistically depicts their traumatic experiences. When therapists encourage such realistic play, clients are gradually exposed to traumatic memories that might otherwise be avoided or suppressed [23]. Moreover, regarding the therapy component of trauma narration, playing out one’s experiences might help children more comfortably express the details of highly traumatic experiences, while also showing emotions and thoughts that can later be processed by the therapist [23].

Third, with Vil Du?!, children are in control of showing their experiences. This control is of particular importance in the context of CSA, in which children were not in control of the situation, and can help them regain a sense of control over their lives [24]. Moreover, children cannot achieve progress in therapy when they feel out of control [25]. With Vil Du?!, children can decide what they will show to the therapist, and they have control over stopping or pausing the game with the *Time-Out* button. Vil Du?! also puts children in control because the use of icons can have various connotations. For example, moving the hand icon over the back of the character can mean a pat on the back, stroking the back, or a slap on the back, depending on the context the child created or on verbal explanations provided by the child. Thus, it is not the game that guides how to interpret play; it is the player who controls the meaning of the interaction. The nondirective nature of Vil Du?! might be helpful in therapy for restructuring incorrect and maladaptive thoughts, because nondirective play can be considered as a restructuring process of one’s thoughts [26].

Fourth, Vil Du?! gives children the opportunity to share and recreate their stories in a safe and nonnormative environment. With Vil Du?!, children can share the story of what happened to them by having one character performing actions on another character. Vil Du?! does not include statements or textual markers that stipulate whether a sexual relationship or action is right or wrong. As such, the game does not demonize the players or their actions, which may create a safe environment for children to open up about their sexual experiences. Normative statements about the inappropriateness of certain sexual behaviors and how children should behave in certain circumstances could problematize an already difficult CSA experience, which may increase feelings of self-blame and shame [27]. Research also shows that creating a narrative of one’s CSA experiences might be a critical mechanism for producing positive outcomes after CSA [28]. In addition, children themselves often mentioned that creating a narrative of the sexual abuse by drawing or writing was specifically the most helpful part of therapy [28,29]. Stories might enable children to create a mental map of events and ideas and revisit them as and when the stories are narrated or remembered again [30]. In addition, games in which children are invited to create or tell a story have been found to help children express themselves safely, because such games aid children in absorbing complex concepts through play [31] as well as allowing children to express their feelings through an indirect medium [26].

### Acceptability of Vil Du?! for its Users

Next, to evaluate the possible working elements of Vil Du?!, it is also important to evaluate the acceptability of the game for its users [17]. Acceptability refers to how intended individual users react to an intervention or tool [32]. Two users are relevant in the context of therapy—the therapist and the client. The therapist’s acceptability of a tool is important in that it determines whether therapists will use the tool. Relevant in this regard is how the therapist feels about the intervention in terms of its benefits and limitations (ie, affective attitude [33]). Client acceptability is clinically important as, if clients prematurely drop out of therapy because they are, for example, dissatisfied with how the therapy is being delivered, therapy cannot be completed and symptoms may persist unnecessarily [34]. Relevant indicators of the acceptability of Vil Du?! by clients might be the emotions, tension, and dissociation (ie, detachment from reality) experienced by clients during the use of Vil Du?! Such experiences are predictive of treatment outcome and adherence [35,36] and can thus provide preliminary information as to whether Vil Du?! is acceptable for clients.

### Goal of This Study

Vil Du?! might be a valuable tool for therapy, but the use of the game in therapy is still in its infancy. For instance, a systematic user manual for Vil Du?! is currently being developed. In addition, therapists are still exploring how Vil Du?! could be used in therapy. To evaluate a tool in such an early phase, an exploratory evaluation among therapists seems most appropriate as it offers more flexibility than rigorous effect studies [17]. Such early evaluations can provide valuable indications of the possible clinical impact of new tools, as well as the acceptability of a tool by its users [37]. This information can subsequently be used for further development or refinement of a tool [17]. Therefore, our mixed-methods study aims to provide an exploratory evaluation of Vil Du?! by answering two research questions. First, which working elements can be identified in how Vil Du?! is used by therapists? We examined the following working elements specific to Vil Du?!: no need to talk; functions as a *third party in the room* or no face-to-face communication necessary; puts the child in control; and playful, safe, and nonnormative environment. Second, how do therapists evaluate the use of Vil Du?! in psychotherapy for CSA? We examined the benefits and limitations of Vil Du?! identified by therapists, as well as therapists’ views of the emotions, tension, and dissociation (ie, detachment from reality) their clients experienced during the use of Vil Du?!

## Methods

### Participants

A total of 21 therapists using the Vil Du?! app at the time of the study were contacted personally, as well as via email, to fill out a web-based questionnaire every time they used Vil Du?! with a client. We could identify these therapists as they had received a working license for Vil Du?! from the app developers. The 21 therapists were also asked to forward the invitation to other colleagues who might have used Vil Du?! The therapists completed the web-based questionnaire for 23 clients with CSA experience. The mean age of the clients was 11.38 (SD 3.96; minimum=5, maximum=18) years. The majority of the clients were women (14/23, 61%). Therapists mostly used Vil Du?! in the context of client-centered therapy (13/23, 57%), followed by play therapy (8/23, 35%), and cognitive behavioral therapy (4/23, 17%). None of the therapists used Vil Du?! for psychoanalytic therapy or group therapy. The total number of therapists participating in the web-based questionnaire could not be determined because therapists could fill out the questionnaire anonymously. A total of 12 therapists provided contact information and indicated that they were willing to participate in the interview part of the study. The characteristics of the 10 therapists who actually participated in the interviews are presented in Table 1.

**Table 1:** Characteristics of therapists participating in the interview phase of the study.

Participant ID	Age (years)	Experience in youth care (years)	Therapy with CSA^a^ clients or therapy related to sexuality (hours per week)	Work organization^b^
1	42	20	5	1^c^
2	44	15	20	1
3	42	17	32	1
4	32	12	32	2^d^
5	52	30	32	1
6	47	22	16	1
7	28	5	16	1
8	42	17	32	3^e^
9	62	10	24	1
10	38	17	24	4^f^

^a^CSA: child sexual abuse.

^b^Participants with the same number in this column work for the same organization.

^c^Youth care organization providing contextual care for child sexual abuse victims.

^d^Specialized youth care organization focusing on adolescent sexuality.

^e^Organization providing specialized family care.

^f^Youth care center for psychotrauma and sexual abuse.

### Design and Procedure

We used a mixed-methods triangulation design with quantitative and qualitative data collected and analyzed at approximately the same time. Both types of data were merged and given equal emphasis in the interpretation [38]. Data were collected via web-based questionnaires (including quantitative and qualitative questions) and semistructured qualitative interviews. Through both methods, we derived input from therapists on the working elements of Vil Du?! and how they evaluate its acceptability. For both the web-based questionnaire and the semistructured interviews, questions and topics were based on the possible working elements of Vil Du?! identified in the literature. We also included aspects of user acceptability derived from existing measures [20].

Therapists completed the web-based survey via LimeSurvey (version 3.20, LimeSurvey Project; closed format with links sent to participants). They provided web-based informed consent for their participation at the beginning of the questionnaire. For the duration of the study (June 2019 to June 2020), we invited therapists to complete the questionnaire each time they used Vil Du?! in therapy sessions. The completion rate was 100% (23/23), as each question was mandatory. Participants were able to change their answers using a back button. The survey included 14 questions (displayed in a fixed order), of which 6 were used for this study.

Subsequently, a trained research assistant conducted and audiotaped interviews with a selection of the therapists (n=10) that completed the aforementioned web-based questionnaire. We selected the widest possible range in terms of the sexual abuse experiences of clients, therapy type, and background characteristics of therapists. For the interview part of this study, therapists provided written informed consent before the start of the interview. The duration of the interviews was, on average, 45 minutes.

The Ethics Committee of the Faculty of Social and Behavioural Sciences of Utrecht University approved this study (FETC19-025). Personal information collected via the survey or interviews was stored on a protected university server. Only the first and second authors had access to this server.

### Web-Based Questionnaire

#### Background Characteristics

Therapists first had to fill out some background characteristics of the client (ie, age and gender).

#### Working Elements and Therapist Acceptability of Vil Du?!

Participants answered 1 open question regarding the elements of Vil Du?! they thought were effective in the therapy process (ie, “What are positive elements of using Vil Du?! in therapy? By positive elements, we mean elements of the game that can explain its effects on the therapy process”). This question (and accompanying explanation) is based on the common definition of working elements [17]. Participants also reported on the limitations of using Vil Du?! for either the therapist or the client (ie, “Is there something missing or lacking in the game that limits the use in therapy?”).

#### Client Acceptability

Client acceptability (according to the therapist) with regard to the use of Vil Du?! was assessed with 3 questions on a 5-point Likert scale [20]:

1. “how pleasant/unpleasant they thought the use of Vil Du?! was for the client” (1=very unpleasant and 5=very pleasant).
2. “how helpful they thought the use of Vil Du?! was for the client” (1=very unhelpful and 5=very helpful).
3. “how enjoyable they thought the use of Vil Du?! was for the client” (1=very unenjoyable and 5=very enjoyable).

### Semistructured Interviews

The interviews were structured around a topic list, including the following topics, and were discussed in more detail than in the web-based questionnaire.

1. What are the differences between using Vil Du?! and not using Vil Du?! in the treatment process or outcomes, and to which elements of Vil Du?! these differences could be attributed (ie, working elements);
2. therapist acceptability (eg, limitations, benefits); and
3. client acceptability (eg, emotions, stress levels, and entertainment value).

### Analyses

#### Web-Based Questionnaire

SPSS version 24 (IBM Corp) was used to summarize and describe the qualitative and quantitative data from the web-based questionnaires. Frequencies were computed to determine the percentage of questionnaires in which the working element of Vil Du?! was identified for a specific client. Frequencies were also computed to determine the percentage of questionnaires in which a certain limitation of Vil Du?! was mentioned for clients of therapists. Descriptive statistics (mean and SD) were computed to summarize the acceptability of Vil Du?! for clients.

#### Interviews and Open Questions From the Web-Based Questionnaire

The stepwise guidelines outlined by Zhang and Wildemuth [39] were followed to increase the efficiency, repeatability, and transparency of our qualitative data analysis of the interview data in NVivo (QSR International). In step 1, we transcribed the answers to all questions literally. Observations during the interview (eg, sounds and pauses) were not coded, because they were not of interest to the research questions. In step 2, we defined the unit of analysis as themes; that is, the working elements of Vil Du?! and the experiences of therapists and clients using Vil Du?! In step 3, a coding scheme was developed, consisting of categories related to the working elements (eg, no face-to-face communication necessary, no need to talk, and child in control), benefits, and limitations of Vil Du?! (the coding scheme is available upon request from the authors). In step 4, the first (JJE) and second author (HT) tested the coding scheme on a sample of text (ie, 2 pages of text selected from a total of 4 interviews) to discover unclarities in the coding scheme. These issues have been discussed and resolved. In step 5, all text was coded by the first author while adding new categories to the coding scheme when necessary. For step 6, a set of randomly selected text fragments (20% of the total number of text fragments) was coded by both the first and second authors. Differences in coding were discussed until a consensus was reached. Changes were made to the coding scheme when necessary. The first author recoded the other 80% of the transcripts on the basis of this changed coding scheme. In step 7, we explored the properties and dimensions of the categories as well as the relations between the categories in the full range of data. We merged categories that reflected the same content. We also specified category names based on the content included in a certain category. Finally, we separated the single categories into multiple categories when a category contained different types of information. Answers to the open questions of the web-based questionnaire were coded using the same codes as the interview data.

#### Merging Quantitative and Qualitative Data From Web-Based Questionnaires and Interviews

For all instances in which data from interviews were merged with data from the web-based questionnaires, data from the interviews were used to further elaborate on the often short answers that were given in the web-based questionnaire.

For aim 1 of this study about the working elements of Vil Du?!, data from the open question about the working elements of the web-based survey were merged with the data from the interviews. More specifically, the percentage of questionnaires that mentioned a certain working element for a client was combined with the percentage of therapists who mentioned the same working element in the interview.

For aim 2 of the study regarding therapists’ evaluations of Vil Du?!, the interviews were used to determine the benefits according to the therapists. In addition, for the limitations, the percentage of questionnaires that mentioned a certain limitation was combined with the percentage of therapists who mentioned the same limitation in the interview. Finally, for client acceptability, quantitative client satisfaction data from the web survey were combined with the percentage of therapists that described in the interviews the expression of certain emotions or levels of tension in their clients.

## Results

### Working Elements of Vil Du?!

Table 2 lists the working elements of Vil Du?! that could be identified in the questionnaires and interviews with therapists. In 30% (7/23) of the questionnaires and in 90% (9/10) of the interviews, therapists recounted experiences in which the child was in control in the game. Sometimes, control was inherent to the game. For instance, clients had control over the use of the Time-Out button. They also had control over what to show and what not to show to the therapist. Finally, they had control over the interpretation of the icons (eg, by moving the hand icon over the back of the character, they could indicate a pat on the pack, stroking the back, or a slap on the back). At other times, the therapist created the control in the game environment. For instance, some therapists invited their clients to choose characters or start up the game. Other therapists gave clients control over the choice to use the game or just talk about CSA experiences.

Most therapists (in 8/23, 35% of the questionnaires and 8/10, 80% of the interviews) further recounted that the game reduced barriers to disclosing CSA experiences for their clients. For example, one therapist mentioned, “I noticed that the tablet helped her to disclose and also a bit to break the ice.” Related to lowering barriers to disclosure, most therapists (in 8/23, 35% of the questionnaires and 9/10, 90% of the interviews) explicitly mentioned that the game could function as a *third party in the room* by creating emotional and physical distance between the therapist, the client, and the client’s CSA experiences. According to a therapist, a client described the emotional distance as follows:

It is very strange, it [the character] is not me, but yet it is me, and I never thought I could tell this much about it.

Regarding the physical distance, a therapist reported as follows:

It is not face-to-face, it is not direct, you talk in a triangle. You talk about something [the tablet] that is on the table and about that what has happened, which creates distance.

**Table 2:** Questionnaire and interview data about the working elements of Vil Du?!

Working elements		Questionnaires (n=23), n (%)	Interviews (n=10), n (%)
Puts child in control		7 (30)	9 (90)
**Reduces barriers to disclosure**		8 (35)	8 (80)
	By creating a third party in the room	8 (35)	9 (90)
	Because there is no need to talk	8 (35)	7 (70)
Congruence with children’s digital experience		—^a^	7 (70)
Playful environment		—	6 (60)
Safe and nonnormative environment		2 (9)	3 (30)
Interactive		—	1 (10)

^a^Not available.

Another working element related to reducing barriers to disclosure was that clients do not need to talk when using the game, which was brought up by therapists in 70% (7/10) of the interviews and in 35% (8/23) of the questionnaires. As one therapist recounted, “Some children find it really difficult to find the words and then it [Vil Du?!] is a very nice tool to use.” Another therapist explained, “‘Show me’ might be easier than ‘tell me’.”

In 70% (7/10) of the interviews, therapists also described that the game was congruent with children’s experience in the digital world. For instance, one therapist mentioned: “They think the app is interesting. It is, of course, very much targeting their digital experience.”

The playful environment of the game was mentioned by therapists in 60% (6/10) of the interviews as a working element. For example, one therapist explained, “What I really like about the game is that in a playful manner you can make very difficult or shameful experiences discussable.”

In 30% (3/10) of the interviews and 9% (2/23) of the questionnaires, therapists brought up the safe and nonnormative environment of the game. As an example, one therapist explained:

In that sense I really liked it and especially that it was very safe for him to tell his story [...], but also without judgement. That screen does not judge.

Finally, in 10% (1/10) of the interviews, the interactive nature of the game was mentioned as a working element. More specifically, the two characters in Vil Du?! can interact with each other, and both the therapist and the client have the possibility to perform actions on the characters and respond to these actions.

### Therapists’ Evaluation of Vil Du?!

#### Therapist Acceptability: Benefits and Limitations

In terms of benefits, time efficiency was mentioned most by therapists in the interviews (8/10, 80%). As an example, one therapist explained: “The benefit is in the rapidness. You are there faster, the thing you want to talk about.” Interestingly, one therapist reported the rapidness of the game as a possible disadvantage, at least if you were not prepared:

If I use it I need to be very alert, very alert. Watch the non-verbal communication [...]. You need to sort of pull out all the stops and ‘am I not missing something?’ [...] But it is, it is not a slow thing. It is very fast. So, if somebody who is going to work with it, who has not used it before, I think ‘pay attention’!

Further, in 50% (5/10) of the interviews, therapists mentioned the benefit that by using Vil Du?! you could obtain more information and details about the client’s CSA experience than without the game. Benefits that were only mentioned in 10% (1/10) to 20% (2/10) of the interviews were as follows: better attention of the client during disclosure of CSA experiences, the possibility to stop the game immediately with the Time-Out button when necessary, the fact that the game is highly structured, the user-friendliness and accessibility of Vil Du?!, and that Vil Du?! is useful for many treatment components. However, of 10 therapists, 1 (10%) thought it was difficult to envision how the game could be used for other treatment components than creating a narrative of the clients’ sexual abuse experiences.

The most mentioned limitation by therapists in 90% (9/10) of interviews and 57% (13/23) of questionnaires was that the game lacks complexity in surroundings and in the characters’ movement, dimensionality, emotion expression, and appearance. For example, one therapist explained that the game lacked complexity in:

the surroundings. For instance, then we went outside, then inside, then to school [...]. So, that it can become more of a story instead of one incident. That fits with our clients, that is, rarely one incident or one place.

Regarding the characters, one therapist reported as follows:

It would be nice if the characters could move a bit more. At least standing, sitting, and lying down. I noticed with the girl I used it for the first time, the girl with intellectual difficulties, that it was very difficult for her, because it always happened on his bed and he was lying down. So, it was very difficult for her to show what happened.

**Figure 4:**
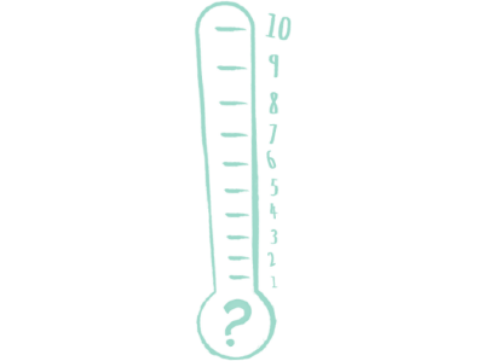
The feeling thermometer in Vil Du?!

Other limitations were only brought up by a few therapists in the interviews and questionnaires. For instance, that it was difficult to visualize masturbation, penetration, or an erection, which was mentioned in 22% (5/23) of the questionnaires and 30% (3/10) of the interviews. In addition, the feeling thermometer (currently a static picture; Figure 4) cannot be manipulated by the client to indicate their levels of distress during the use of Vil Du?! (3/23, 13% of questionnaires and 3/10, 30% of interviews). Two therapists further mentioned in the interviews that the game could be boring or childish for older clients.

Finally, some limitations were recounted by single therapists only in either the questionnaire or the interview; therefore, these limitations might be subjective. For example, the game included limited content for psychoeducation or normalizing sexual behavior (mentioned in the questionnaire). Another therapist mentioned technical problems with the game during the interview. An additional limitation was that it was not possible to change the explicitness of the icons (interviews). Relatedly, the vagina icon might not be clear enough for young children (interviews). Finally, one therapist mentioned that the game is difficult to use for older therapists (interviews).

#### Client Acceptability: Emotion Expression, Level of Tension, and Dissociation According to Therapists

Questionnaire data showed that all aspects of client satisfaction (according to therapists) yielded mean scores above the neutral midpoint of the scale. The highest scores were given for the usefulness of Vil Du?! for the client’s therapy progress. Therapists thought the use of Vil Du?! was between somewhat and highly useful for their clients (mean 4.13, SD 1.14). Therapists also thought the use of Vil Du?! was, as far as possible in the context of CSA, somewhat of a pleasant experience for their clients (mean 3.57, SD 1.47) as well as a bit enjoyable (mean 3.30, SD 1.40).

Similarly, during the interviews, most therapists (9/10, 90%) recounted their clients’ positive emotions when using Vil Du?! or shortly after using Vil Du?! Examples of such positive emotions are fun, joy, happiness, and pride. In 50% (5/10) of the interviews, therapists also described clients who expressed negative emotions, such as fright, irritation, clenching, and anger. Finally, one therapist described 2 clients who were rather neutral in terms of their emotional expression.

With regard to clients’ level of tension, in 70% (7/10) of the interviews, therapists described clients experienced lower or shorter bouts of tension when using Vil Du?! than by simply talking about their experiences. In 40% (4/10) of the interviews, therapists mentioned clients who experienced high levels of tension or signs of dissociation, but they did not link these experiences specifically to the use of Vil Du?! One therapist described the possibility that Vil Du?! elicits higher levels of tension because of its explicit nature, but the presence of the Time-Out button makes tension manageable.

## Discussion

### Principal Findings

The goal of this study is 2-fold. The first aim is to identify the working elements in how Vil Du?! was used by therapists. By combining data from questionnaires and semistructured interviews, we found that therapists identified several working elements of Vil Du?! More specifically, the therapists mentioned that Vil Du?! puts the child in control. They also described that the game reduces barriers to disclosure as there is no need to talk and no need for eye contact with the therapist. Furthermore, they thought that the game was congruent with the children’s digital experience. In addition, they mentioned that the game presents a playful as well as a safe and nonnormative environment. Finally, they mentioned the interactivity of the game as a working mechanism.

The second aim is to investigate how therapists evaluated the use of Vil Du?! in psychotherapy for CSA. With regard to this aim, Vil Du?! was generally evaluated more positively than negatively by therapists, indicating the acceptability of the tool by therapists. Therapists mentioned benefits such as time efficiency and the ability to obtain more information and details about clients’ CSA experiences. Limitations were more technical in nature, such as the lack of complexity of surroundings and characters in the game environment, certain sexual behaviors that could not be visualized properly, and a feeling thermometer that could not be manipulated. With regard to client acceptability, according to therapists, most clients experienced less tension and more positive (or neutral) emotions than negative emotions when using Vil Du?! to discuss CSA experiences.

### Working Elements of Vil Du?!

A working element of Vil Du?! that most therapists identified was that the game reduced the barriers to disclosure. This is not surprising considering that many individuals having experienced CSA are faced with barriers to disclosure, such as lack of verbal abilities, shame, guilt, avoidance, and tension. Talking about CSA experiences is known to be difficult for these reasons (among others) [3]. Several other working elements of the game could explain why and how the game reduces which specific barriers. For example, the element that it is not necessary to talk about CSA experiences might specifically reduce the barrier with regard to lack of verbal abilities or vocabulary. Furthermore, the game does not require eye contact between therapist and client and, as such, functions as a *third party in the room* [20], allowing children to express their feelings through an indirect medium [26]. Eye contact might be particularly intolerable for clients who experience guilt, shame, and avoidance [40]; thus, with Vil Du?!, sharing their shameful experiences could be more tolerable. Moreover, Vil Du?! presents children with a safe and nonnormative environment that does not dictate right or wrong, which may reduce feelings of self-blame and shame [1,27]. In addition, the game presents the client with a playful environment, and play is known to reduce tension and stress [9].

The play element of the Vil Du?! app might have an effect on several components of the therapy. For example, individuals with CSA experience often have incorrect or maladaptive thoughts related to abuse experiences (eg, “I could have prevented the abuse”) that need to be restructured in therapy. The type of nondirective play that is possible in Vil Du?! itself might be considered a restructuring process [26]. Playing out one’s experience through Vil Du?! might also help to make thoughts related to one’s CSA experiences explicit [21,22]. These explicit thoughts can be processed or restructured in successive therapy sessions. In addition, play that realistically depicts children’s traumatic experiences, which might be possible with the explicit icons and the characters in Vil Du?!, can be used to achieve gradual exposure of clients to traumatic memories that might otherwise be avoided or suppressed [23]. Finally, playing out one’s experiences through Vil Du?! might be the first step toward creating a narrative of one’s CSA experience [28]. Creating such a narrative is most often done by drawing or writing and is experienced by children as the most helpful part of therapy [28,29].

Therapists also identified other working elements not directly linked to the barriers to disclosure. The first was that the child was in control in the game. Therapists acknowledged the importance of children regaining control over their CSA experiences. Giving children control in therapy sessions is an important factor for achieving progress in therapy [25] and helps children regain a sense of control over their lives [24]. Giving children control through Vil Du?! could be accomplished by aspects inherent to the game, such as the client’s use of the Time-Out button or control over what to show to the therapist. Therapists also actively invited clients to take control in operating the game. Giving the clients control in operating the game fits with children’s experience and frequent involvement with apps and video games [41].

Congruence of Vil Du?! with children’s digital experience was another working element that was mentioned by the therapists. Vil Du?! was played on the 2 tablets. Especially for young children, the touchscreen technology of tablets offers a mode of interactive experience that is congruent with children’s natural ways of learning and exploring (ie, touch, repeat, trial, and error) [42]. In addition, for adolescents, the use of hand-held devices, such as tablets, mirrors the way they are used to communicating with their peers through social media [43].

Finally, one therapist mentioned the interactive nature of the game as a working element. Vil Du?! enables interactivity between the therapist and the client. Both therapist and client *play* the game on their own tablet. The tablets are synchronized to each other, so actions performed on one screen are also visible on the other. More specifically, both the therapist and client can represent a character in Vil Du?! and each character can perform actions on the other character, as well as respond to the actions of the other character. Researchers have suggested that interactivity in games “can bring a reciprocal effect to other participants of the communication process by turn-taking, feedback, and choice behavior” [44]. Instant reactions from another player might create quick feedback loops to provoke deeper thinking and learning, as well as making sense of previous experiences [45].

### Acceptability of Vil Du?! by Therapists and Clients

In terms of the evaluation of Vil Du?! by therapists, findings show that the game might be acceptable for therapists as well as clients. For therapists, the game increased the efficiency of the therapy process and simultaneously led to the discovery of more details and information about the clients’ CSA experiences. The time efficiency of the use of serious games in psychotherapy for children has also been suggested by other scholars [46,47]. Time efficiency of Vil Du?! could be because of the visual nature (ie, showing is faster than telling) or because of the safe environment Vil Du?! created by allowing children to express their feelings through an indirect medium [26]. The large amount of details and information gathered with Vil Du?! is similar to research showing that children provided more detail about emotionally laden events when they could draw or re-enact the event compared with when they were simply asked to tell [48]. An explanation could be that the icons in Vil Du?! may have provided the client with additional and effective retrieval cues for their CSA-related memories. According to therapists, the most important benefit for clients of discussing CSA experiences with Vil Du?! is a possible reduction in negative emotions and tension compared with just talking about one’s experiences. Less negative experiences during the treatment process are associated with more favorable treatment outcomes and better treatment adherence [35,36].

These benefits seem to outweigh the limitations of the game mentioned with regard to the lack of complexity of characters, surroundings, and icons. These limitations were mainly technical in nature and could be incorporated into an updated version of the game. Other limitations might be specific to certain therapists or clients. For example, as mentioned by one older therapist, the game might be difficult to use mainly for older therapists, because another younger therapist recounted the user-friendliness of the game, particularly as a benefit. For older therapists or less technically skilled therapists, a user manual for Vil Du?! is particularly pertinent and could increase usability.

### Limitations of the Study and Directions for Future Research

An important limitation of this study was that we focused mainly on the therapists’ perspective on the use of Vil Du?!, even though we also asked therapists about their clients’ experiences. However, clients themselves might evaluate the use of the game in therapy differently. At the start of the study, we aimed to include the clients’ perspective more by videotaping and coding therapy sessions in which Vil Du?! was used. Even though this part of the study was ethically approved, we were unable to obtain consent from enough clients and their parents to include this method in our study. Future research could include clients’ perspectives by asking clients to fill out short, age-appropriate evaluation questionnaires following the use of a serious game in psychotherapy.

A second limitation relates to the questionnaire we used to assess client acceptability, which was composed of only 3 items. Although these items have been used in previous research [20], 3 items may have been limited in their ability to fully assess client acceptability. Future evaluations of Vil Du?! could use more extensive measures of client acceptability, such as the Child Evaluation Inventory [49].

A third limitation is that we were not able to include therapists that represented a wide range in terms of the therapy type they used and the organization they worked for. The majority of participating therapists were working for the same organization and, thus, with the same treatment protocols. This may have limited the ways in which therapists used the Vil Du?! app and subsequently, the possible working elements that were identified.

It is important to note that there were some differences in the working elements and limitations identified in the questionnaire data and the interview data. Overall, the percentages of working elements and limitations mentioned by therapists were lower in the questionnaires than in the interviews. These differences could be because of the different formats of the 2 methods. In the semistructured interviews, therapists could freely and extensively discuss their experiences with using Vil Du?! In the web-based questionnaire, therapists had to write down their experiences and, consequently, their answers were generally short. However, the relative importance of specific working elements and limitations were very similar across methods. The working elements and limitations that were most often mentioned in the questionnaires were also the ones most often mentioned in the interviews.

Finally, we identified several working elements of how Vil Du?! was used by therapists. However, we could only speculate about the reasons for the effectiveness of these elements in the treatment of CSA victims. Future experimental or longitudinal research should examine the effectiveness of incorporating Vil Du?! in CSA treatment. These studies could also examine the specific working elements underlying the effects Vil Du?! may have on the therapy process and client outcomes. However, to take this next step in evaluating the effects of Vil Du?!, the findings of this study will be incorporated into a user manual for Vil Du?! With this manual, therapists can use the game in a more systematic way (access to the manual and Vil Du?! can be arranged through the authors).

### Conclusions

This exploratory evaluation of Vil Du?! provided promising results for the incorporation of serious games in therapy sessions with children and adolescents. The case study on Vil Du?! clarifies how a game specifically designed to help children open up about CSA experiences might improve psychotherapy for child and adolescent CSA victims. Using Vil Du?! appears to be time efficient, and the game appears to make the treatment process less confronting and difficult for the client. In this study, several working elements of Vil Du?! were identified, which could explain possible improvements in the therapeutic process. First, Vil Du?! enables clients to regain control over their CSA experiences. Second, the game offers a nonverbal communication tool that empowers clients with additional vocabulary (eg, interactions on the screen). Disclosure of clients’ experiences is furthermore enabled by creating a safe environment in which the client can work on their own tablet and does not need to make eye contact with the therapist. The increased amount of vocabulary, control, and safety might result in a significant time reduction in psychotherapy. This benefits both the client and health care system in general. Rigorous experimental effect studies are now necessary to test whether implementing the serious game Vil Du?! in therapy is effective in reducing barriers to disclosing CSA experiences and in regaining control over these experiences.

## Abbreviations

CSA: child sexual abuse
